# Preparation of Carboxymethyl Cellulose-*g*- Poly(acrylamide)/Attapulgite Porous Monolith With an Eco-Friendly Pickering-MIPE Template for Ce(III) and Gd(III) Adsorption

**DOI:** 10.3389/fchem.2020.00398

**Published:** 2020-05-25

**Authors:** Feng Wang, Yongfeng Zhu, Aiqin Wang

**Affiliations:** ^1^Key Laboratory of Clay Mineral Applied Research of Gansu Province, Center of Eco-material and Green Chemistry, Lanzhou Institute of Chemical Physics, Chinese Academy of Sciences, Lanzhou, China; ^2^Qinzhou Key Laboratory of Biowaste Resources for Selenium-Enriched Functional Utilization, College of Petroleum and Chemical Engineering, Beibu Gulf University, Qinzhou, China

**Keywords:** porous materials, pickering emulsions, attapulgite, adsorption, rare earth elements

## Abstract

Due to their high specific surface and metal-binding functional groups in their crosslinked polymeric networks, monolithic materials incorporating a porous structure have been considered one of the most efficient kinds of adsorbents for rare earth element recovery. Herein, a facile and novel monolithic multi-porous carboxymethyl cellulose-*g*-poly(acrylamide)/attapulgite was synthesized by free radical polymerization via green vegetable oil-in-water Pickering medium internal phase emulsion (O/W Pickering-MIPEs), which was synergically stabilized by attapulgite and tween-20. The homogenizer rotation speed and time were investigated to form stable Pickering-MIPEs. The effects of different types of oil phase on the formation of Pickering-MIPEs were investigated with stability tests and rheological characterization. The structure and composition of the porous material when prepared with eight kinds of vegetable oil were characterized by FTIR and SEM. The results indicate that the obtained materials, which have abundant interconnected porosity, are comparable to those fabricated with Pickering-HIPE templates. The adsorption experiment demonstrated that the prepared materials have a fast capture rate and high adsorption capacities for Ce(III) and Gd(III), respectively. The saturation adsorption capacities for Ce(III) and Gd(III) are 205.48 and 216.73 mg/g, respectively, which can be reached within 30 min. Moreover, the monolithic materials exhibit excellent regeneration ability and reusability. This work provides a feasible and eco-friendly pathway for the construction of a multi-porous adsorbent for adsorption and separation applications.

**Graphical Abstract:**
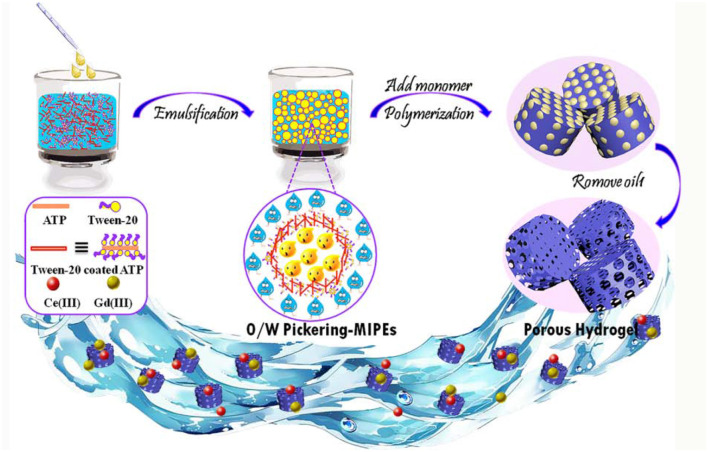
The preparation of porous monolith with Pickering-MIPE template and it's application for Ce(III) and Gd(III) adsorption.

## Introduction

Rare earth elements (REE) are critical components in special functional materials for industrial applications as well as in cutting-edge technology fields, such as biomedical engineering, information storage, communications, wind turbines, spaceflight, nuclear energy, and military applications (Saravanan et al., 2013; Ogata et al., 2015; Anastopoulos et al., 2016; Rajendran et al., 2016; Zhao et al., 2016, 2019; Wang et al., 2017a). China possesses more than 90% of the global potential reserves (Survey, 2014; Ogata et al., 2015). Recently, the adsorption and recovery of REEs from aqueous solutions has become a significant issue owing to their significance (Voßenkaul et al., 2015; Alshameri et al., 2019), and many technologies are being developed to enrich REEs, including solvent extraction (Hou et al., 2013; Schirhagl, 2014), adsorption (Maranescu et al., 2019; Mondal et al., 2019; Yang et al., 2019), ion-exchange (Moldoveanu and Papangelakis, 2012; Page et al., 2019), and co-precipitation (Chatterjee et al., 2009). Among them, adsorption is considered one of the most promising methods for the removal of REEs due to its simple operation, easy separation, high efficiency, and reusability (Ogata et al., 2015; Wang et al., 2017a).

At present, various adsorbent materials have been employed to adsorb REEs from aqueous solutions including red clay (Gładysz-Płaska et al., 2014), hydrogel porous materials (Zhu et al., 2016a), carbon materials (Babu et al., 2018), silica gels (Ogata et al., 2015), marine sediments (Liatsou et al., 2015), and biopolymer microcapsules (Delrish et al., 2014). Among various adsorbents used, hydrogel porous materials are one of the most efficient types of adsorbents due to their superhydrophilic features and the metal-binding functional groups of the cross-linked polymeric networks (Ren et al., 2016). Moreover, hydrogel porous materials can be designed and tailored by various functional groups to provide them with specific properties, and the resulting functional materials are favorable for a stable adsorption process. Furthermore, the adsorption properties of adsorbents depend on their functional groups and specific surface area. A high specific surface favors the exposure of the functional groups, improving the probability of reaction between adsorbates and adsorbents. Thus, incorporating a porous structure into adsorbents can provide a high specific surface area for adsorbents to effectively remove REE, and the porous structure also has the benefits that it reduces transmission resistance and enhances the adsorption activity of adsorbents to adsorbates (Zhu et al., 2016b). Therefore, it is indispensable to develop functional hydrogel materials with a porous structure.

The soft template method is a common method of constructing materials with ordered and disordered porous matrices (Jiaxi et al., 2015). In this method, oil-in-water Pickering high internal phase emulsions (O/W Pickering HIPEs) containing more than 74% internal phase is considered the most effective pathway for the preparation of hydrogels with well-defined porosity (Zhu et al., 2016a,b). This technology involves the polymerization of monomers in the continuous phase and the removal of the dispersed phase, which could create a solid foam material (polyHIPE) with an interconnected porous structure (Ma et al., 2014; Oh et al., 2014). Interestingly, the interconnectivity and pore structure of polyHIPEs can be easily tailored by varying the parameters of O/W Pickering HIPEs (Ikem et al., 2010a; Ye et al., 2013). Therefore, large numbers of adsorbents with interconnected structures have been successfully prepared by using O/W Pickering HIPEs (Yi et al., 2016), and the as-prepared adsorbents exhibited enhanced adsorption capacity to model pollutants due to abundant porosity and high permeability (Zhang et al., 2016).

However, the O/W Pickering HIPEs have some obvious drawbacks. For example, this method often requires more than 74% of an organic solvent (such as liquid paraffin, toluene, *p*-xylene, or *n*-hexane) as the dispersion phase, and needing a large amount of these organic solvents greatly increases the preparation cost; in addition, these are harmful to the environment and human health. It is thus desirable to decrease the oil phase fraction and substitute poisonous organic solvent with an eco-friendly oil phase. Compared with Pickering-HIPEs, the formation of Pickering-medium internal phase emulsions (Pickering-MIPEs) requires less of the dispersion phase. Therefore, in this research, porous materials with medium internal phase emulsions have been fabricated by using cheap vegetable oil as the dispersion phase. In addition, particles with appropriate wettability are a necessary condition for preparing a stable O/W Pickering emulsion. In order to achieve the necessary wettability, solid particles [such as inorganic particles (Gudarzi and Sharif, 2011), polymer micelles (Yang et al., 2016), colloidal particles (Gautier et al., 2007), and carbon materials (Sullivan and Kilpatrick, 2002)] usually need to be modified by chemical and physical processes, but it is inevitable that this will result in waste energy and an increase of production cost. For these reasons, natural particles that require no further modification are considered excellent stabilizers for the construction of interconnected porous materials. Due to their plentiful hydroxyl groups and negative charge, clay minerals are highly hydrophilic and present the potential for use in the formation of an O/W emulsion as stable particles without any further modification.

In this study, natural attapulgite (ATP) was employed as the stable particle, tween-20 (T-20) served as the co-stabilizer, and vegetable oil was selected as the disperse phase to form eco-friendly O/W Pickering-MIPEs for the construction of porous materials. In order to form stable Pickering-MIPEs, the effects of the type of oil phase, the homogenizer rotation speed, and time on the formation of Pickering-MIPEs were investigated. Sodium carboxymethyl cellulose (CMC) has two different end groups: one has a chemically reducing functionality (a hemiacetal unit), and the other has a pendant hydroxyl group that can be used to react with other monomers (Klemm et al., 2005). Moreover, the groups on CMC were found to be effective for the removal of metal ions from water (Sharma et al., 2017; Klemm et al., 2018). Thus, in this research, CMC was employed as the grafting backbone onto which to graft acrylamide (AM), which was used as the functional monomer due to its ability to combine with metals and its reactivity. The adsorption properties of the obtained porous cellulose-*g*- poly(acrylamide)/attapulgite materials for Ce(III) and Gd(III) from aqueous solution was also studied.

## Experimental Section

### Materials

CMC (CP, viscosity: 300-800 mpa·s) and AM (CP) were purchased from Shanpu Chemical Factory (Shanghai, China) and used without further treatment. Ammonium persulfate (APS, AR), *N,N,N'N'*-tetramethyl ethylenediamine (TMEDA, AR), Methyl violet (MV), and Methylene blue (MB) were provided by Sinopharm Chemical Reagent Co., Ltd. (Shanghai, China). ATP was obtained from Huangnishan Mine in Xuyi county, Jiangsu province, China, and then ground and passed through a 200-mesh sieve. *N,N'*-methylenebisacrylamide (MBA, CP) was received from Yuanfan Vegetable Additives (Shanghai, China). T-20 (AR) was received from BASF Corporation. 3A molecular sieve 103 was obtained from Molsion Molecular Sieve Co., Ltd. (Shanghai, China). Linseed oil was purchased from Yongfan Trading Co. (Lanzhou, China). Colza oil and soybean oil were purchased from Yihaijiali Arawana Edible Oil Co., China. Peanut oil, sunflower oil, olive oil, and sesame oil were obtained from Shandong Luhua Group Co., Ltd. China. Corn oil was purchased from Cofco Food Marketing Co., Ltd. China. Other reagents were all of analytical grade, and all solutions were prepared with deionized water.

### Preparation of Pickering-MIPEs

T-20 (0.2 g) and ATP (0.5 g) were dissolved into 10 mL deionized water under 3,000 rpm stirring for 1 min, and then various vegetable oils (10 mL) were added into the mixed suspension separately and emulsified with a GJD-B12K homogenizer at certain rpm values for a designed time to form Pickering MIPEs. The type of emulsion was detected by the pendant-drop method with deionized water and *p*-xylene. The emulsion droplet could disperse into deionized water but remained round in *p*-xylene, indicating that the as-prepared Pickering-MIPEs were O/W emulsions. The feed composition of reactants, the codes of the emulsions, and the corresponding droplet diameters are listed in Table 1.

**Table 1:** Feed composition of Pickering MIPE formulations and porous CMC-*g*-PAM/ATP monoliths and their corresponding average droplet and pore sizes.

**Emulsion**	**Speed (rpm)**	**Time (min)**	**Emulsion**	**Speed (rpm)**	**Time (min)**	**Oil**
ES-4000	4,000	10	ES-12000	12,000	10	Linseed oil
ES-6000	6,000	10	ET-1	10,000	1	Linseed oil
ES-8000	8,000	10	ET-3	10,000	3	Linseed oil
ES-4000	4,000	10	ET-5	10,000	5	Linseed oil
ES-10000	10,000	10	ET-7	10,000	7	Linseed oil
ES-11000	11,000	10	ET-9	10,000	9	Linseed oil
**Emulsion**	**D**_**E**_ **(μm)*^a^***	**Materials**	**D**_**M**_ **(μm)*^b^***	**Speed (rpm)**	**Time (min)**	**Oil**
E1	2.54	S1	2.35	10,000	7	Linseed oil
E2	2.48	S2	2.29	10,000	7	Colza oil
E3	2.57	S3	2.39	10,000	7	Peanut oil
E4	2.49	S4	2.32	10,000	7	Sunflower oil
E5	2.52	S5	2.27	10,000	7	Soybean oil
E6	2.58	S6	2.31	10,000	7	Corn oil
E7	2.55	S7	2.28	10,000	7	Olive oil
E8	2.43	S8	2.24	10,000	7	Sesame oil

a *Average diameter of emulsion droplets*.

b *Average pore diameter of materials*.

### Preparation of Porous CMC-g-PAM Monolith

The stable Pickering-MIPEs containing 0.1 g CMC, 0.31 g MBA, 2% T-20, and 5% ATP were prepared by first dissolving CMC and MBA in the continuous phase. After the emulsion was obtained, 1.42 g (2 mmol) AM, 91 mg APS, and 0.1 mL of TMEDA were added into it with rapid stirring for 1 min, and then the prepared mixture was transferred into test tubes, sealed, and immersed in a 65°C water bath for 12 h to complete the polymerization reaction. After that, the porous polymers were sectioned and washed with acetone via Soxhlet extraction for 10 h. In order to activate the porous polymers, they were immersed in 0.5 M NaOH aqueous alcohol solution (V/V, 3/7) for 24 h to transform amide groups into carboxyl groups. The redundant NaOH and undesirable residues were washed away with a water/ethanol (*V*_water_/*V*_alcohol_ = 3/7) solvent, repeatedly, and then the monolithic hydrogels were dehydrated with 3A molecular sieve in absolute ethanol and then dried at 60°C in a vacuum for 8 h. The feed composition of the reactants, the codes of the porous monoliths, and the corresponding pore diameters are also summarized in Table 1. Photographs of the materials are shown in Figure 4.

### Characterization

FTIR spectra were obtained by a Nicolet NEXUS FTIR spectrometer (U.S.A.) in the 4,000–400 cm^−1^ wavenumber region after the samples had been prepared as KBr pellets. The morphologies of the porous monoliths were characterized using a field emission scanning electron microscope (SEM, JSM-6701F, JEOL, Japan) after being coated with gold film. The average pore diameter was obtained by measuring 100 pores using Image-Pro Plus 6.0 software. A Leica DM1000 digital biological microscope equipped with a built-in camera was used to record optical microscopy images of the Pickering-MIPEs.

### Evaluation of Adsorption Performance

Working solutions containing different concentrations of Ce(III) and Gd(III) were prepared by diluting the stock solution of Ce(NO_3_)_3_ or Gd(NO_3_)_3_ using deionized water. The monolithic adsorbents were ground into granular particles with a particle size in the range of 12–40 mesh to conduct the adsorption experiments.

The adsorption process was performed by immersing 20 mg of adsorbent in 25 mL Ce(III) and Gd(III) solutions and shaking with a thermostatic shaker (THZ-98A) at 120 rpm and 30°C for a given time to reach adsorption equilibrium. After that, the monolith was separated from the solution, and the absorbance of solutions was measured by a UV-visible spectrophotometer (UV-3010, HITACHI, Japan) to measure the concentrations of Ce(III) and Gd(III) in the solution. The complexing agents used in this case are chlorophosphonazo and azo arsine for Ce(III) and Cd(III), respectively. The maximum absorbance wavelengths are 666 nm for Ce(III) and 656 nm for Gd(III). The adsorption capacities of the porous monolith for Ce(III) and Gd(III) were calculated according to Equation (1):

(1) $$\begin{array}{r} q_{\text{e}}=\left(C_{0}-C_{\text{e}}\right)\times 0.025/m \end{array}$$

where, *q*_e_ (mg/g) is the amount of Ce(III) or Gd(III) adsorbed by per unit mass of adsorbent and *C*_0_ and *C*_e_ (mg/L) are the concentrations of Ce(III) or Gd(III) before and after adsorption, respectively, 0.025 (L) is the volume of Ce(III) or Gd(III) solution used for adsorption, and *m* (mg) is the mass of adsorbent.

During the experiment, the pH of the solution was adjusted to the required value with 0.1 mol/L HCl or NaOH solutions. The influence of pH value on the adsorption capacities of the porous monolith (S2) was evaluated in rare earth metal solutions with an initial concentration of 200 mg/L and pH values from 1 to 7. The effect of initial concentration on the adsorption capacities of the porous monolith was investigated by adding adsorbent (S2) into solutions with concentrations in the range of 100–400 mg/L for 1 h at the original pH of the solution. The adsorption kinetics was determined by ranging the adsorption duration from 5 to 120 min at the original pH of the solution and with a solution concentration of 200 mg/L. All adsorption experiments were repeated thrice to obtain the average value.

Desorption experiments were conducted to show the recyclability of the prepared porous hydrogel. Typically, 20 mg of the adsorbent (S2) was fully contacted with 25 mL 200 mg/L at 120 rpm and 30°C for 1 h to attain saturation adsorption. The metal-loaded adsorbent was then soaked in 30 mL HCl (0.5 mol/L) solution for 2 h to desorb, and then soaked with 0.5 mol/L NaOH solution and fully washed to neutral with deionized water to activate, after which the adsorbent was used for another adsorption process. The adsorption-desorption cycle was repeated five times to evaluate the reusability of the monolith.

## Results and Discussion

### Preparation of Pickering-MIPEs Stabilized by ATP and T-20

**Scheme 1:**
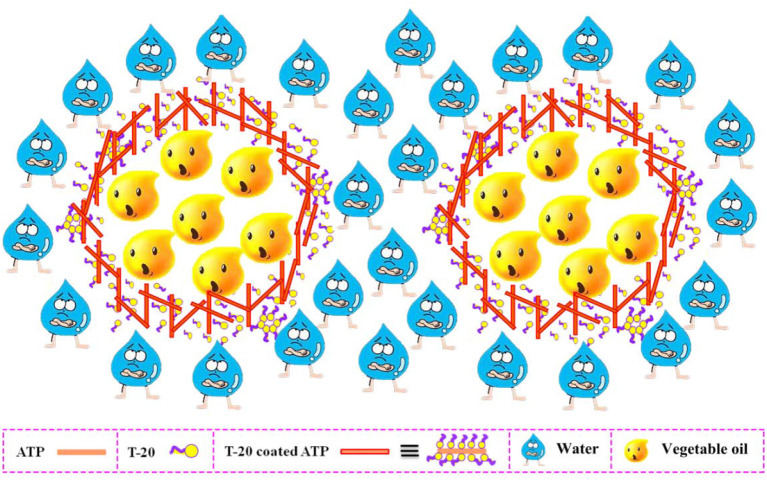
Formation of O/W Pickering-MIPEs stabilized by ATP and T-20.

The eco-friendly O/W Pickering MIPEs were formed by using vegetable oil as the dispersion phase and ATP and T-20 as the stabilizer after vigorous stirring for the designed time. During the stirring progress, T-20 first coated the surface of the ATP and then coated ATP nanorods synergistically with other free surfactants at the oil–water interface, acting as a barrier to prevent the coalescence of oil droplets and to generate a stable emulsion, as shown in Scheme 1.

#### The Effects of Emulsification Speed and Time

**Figure 1:**
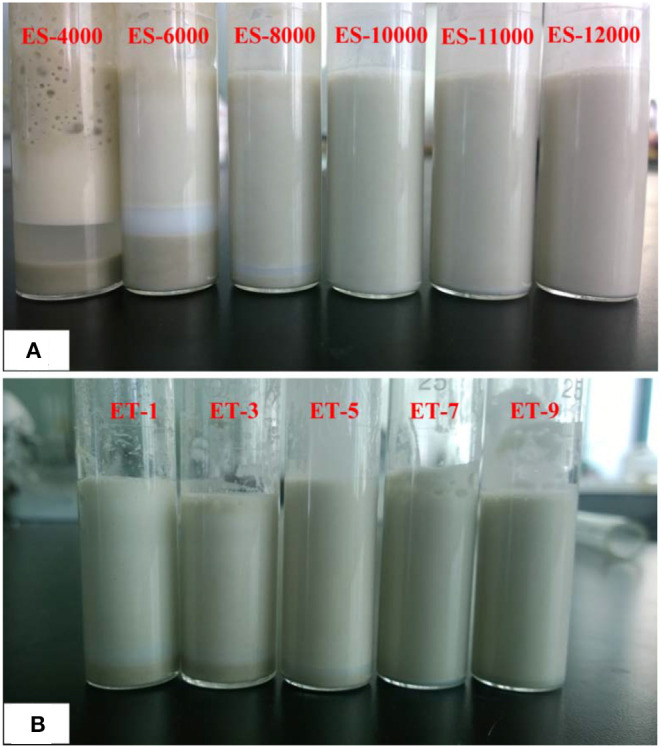
Effects of **(A)** the homogenizer rotation speed and **(B)** time on the formation of Pickering-MIPEs. All of the emulsions were stabilized by 5% ATP and 2% T-20.

The homogenizer rotation speed and time are important factors for obtaining a homogenous emulsion (Colla et al., 2006), and thus the effects of the stirring velocity and time were investigated in this case. The experiment details are listed in Table 1; all the emulsions were stabilized by 5% ATP and 2% T-20. After preparation, the emulsion was kept for a month to observe its stability. As shown in Figure 1, the homogenizer rotation speed had a greater effect on the stability of emulsions compared with emulsification duration (Figure 1A). As the emulsification velocity and shear stress increased, the energy of the emulsion systems also increased to reduce the system energy, and the droplet size of emulsions largely reduced. The increase in the emulsification velocity expanded the contact area of the aqueous and oil phases, allowing more efficient dispersal and breakup of oil droplets in the emulsion and thus greatly enhancing the stability of emulsions. It is worth noting that an excessive emulsification speed introduced a large amount of bubbles into the emulsion. According to Figure 1, the volume of emulsion remained the same after the velocity reached 10,000 rpm, and thus 10,000 rpm was selected for the evaluation of the effect of emulsification duration.

At the same emulsification speed, an increase in the emulsification duration also helped to form a stabilized emulsion. Sufficient emulsification time could enhance the dispersion of the emulsion, decrease the diameter and wall thickness of droplets, and also increase the amount of droplets. However, collisions between droplets due to the mechanical agitation led to their aggregation and the formation of foam at the critical time, and thus it was essential to find the appropriate duration for an emulsion process. As shown in Figure 1B, stabilized emulsions were obtained after being stirred for 7 min, and thus the optimal duration of the emulsion process was 7 min.

#### The Effect of the Oil Phase

**Figure 2:**
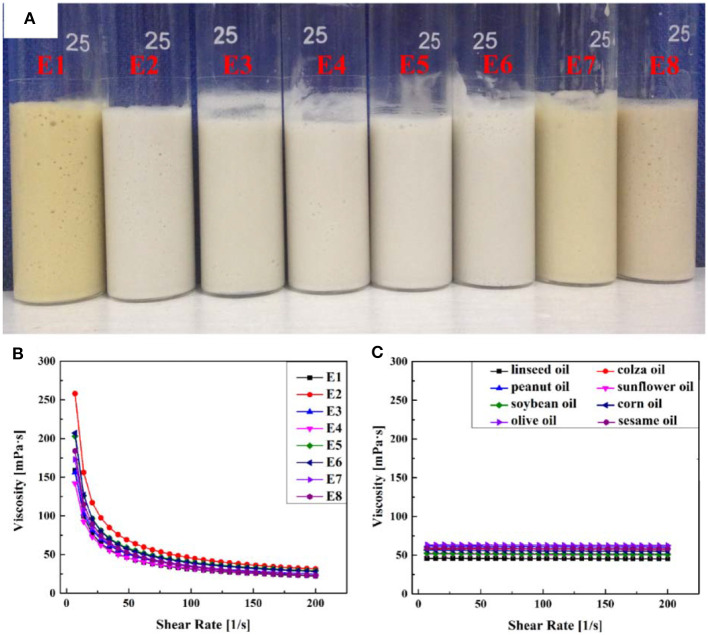
**(A)** Digital photographs of Pickering emulsions prepared with various vegetable oils. Rheological characterization of **(B)** the corresponding Pickering emulsions and **(C)** the different vegetable oils.

**Figure 3:**
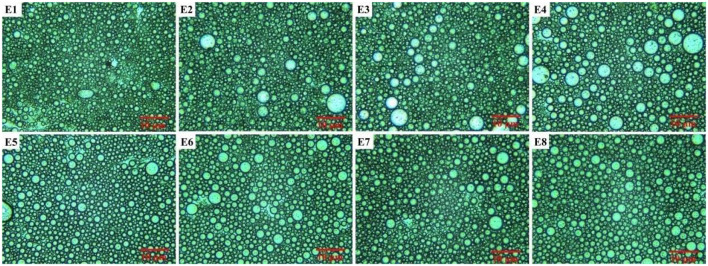
Optical microscope images of Pickering emulsions prepared with different oil phases.

The effect of eight types of vegetable oil on the emulsions was also studied. Digital photographs and rheological characterization of Pickering emulsions with various vegetable oils are shown in Figure 2. As depicted in Figure 2A, the emulsions prepared with eight kinds of vegetable oils maintained good stability for at least 1 month after being agitated at 10,000 rpm for 7 min, and no oil–water separation was observed. The different colors of the eight emulsions might be attributable to the different original colors of the oil phases. A sliding test indicated that the emulsions could stay on a vertical glass tube without any flow, which suggested that the emulsions formed were typical gel emulsions and that the oil drops were closely packed, which was also verified from their optical micrographs (Figure 3). It was also confirmed that the increase in the viscosity of emulsions could inhibit the coalescence of emulsion droplets and improve the stability of emulsion (Ikem et al., 2008). Therefore, a stress-controlled rheometer was used to evaluate the viscosity of the prepared Pickering emulsions. The rheological characterization reveals that there was no significant difference in the viscosity of the eight emulsions (Figure 2B) and the viscosity of the corresponding vegetable oils (Figure 2C).

The optical microscope images of the obtained emulsions are shown in Figure 3. It was found that the droplets of all eight emulsions accumulated together compactly and that the average droplet diameters of the eight emulsion droplets were almost the same (Table 1). The lack of visible difference between the emulsion diameters might be another important reason for the similar viscosity of the emulsions. The above experimental results proved that type of oil did not have a significant influence on the emulsion, and thus all vegetable oils used in this research were suitable for preparing a stable O/W Pickering emulsion.

### Formation of CMC-g-PAM/ATP

**Figure 4:**
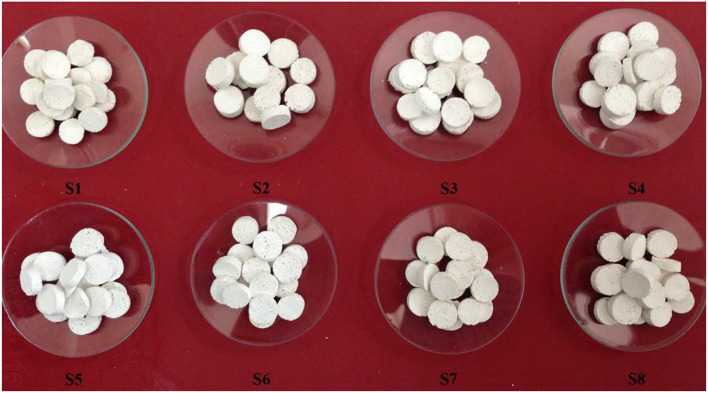
Digital photographs of the porous CMC-*g*-PAM/ATP hydrogels prepared through the use of Pickering-MIPEs with various vegetable oils.

The porous monoliths were obtained by free radical polymerization of the reactants in the continuous phase using APS as the initiator. The decomposition of APS at 65°C induced the CMC to generate macro-radicals, and the monomer of AM was grafted onto CMC. The three-dimensional net structure polymer was then formed in the presence of the crosslinker MBA. After the reaction was finished, the products were Soxhlet extracted with acetone immediately for 10 h to remove the oil phase, and monoliths with porous morphology were observed (Figure 4). This suggested that the incorporation of appropriate ATP and a low dosage of T-20 as a Pickering stabilizer was successful in forming a porous structure inside hydrogels during the polymerization and post-treatment. The elution of the oil phase contributed to the formation of macropores, while the thinner monomer layer between neighboring droplets resulted in open pore throats between neighboring macropores (Xu et al., 2016; Wang et al., 2017b).

**Figure 5:**
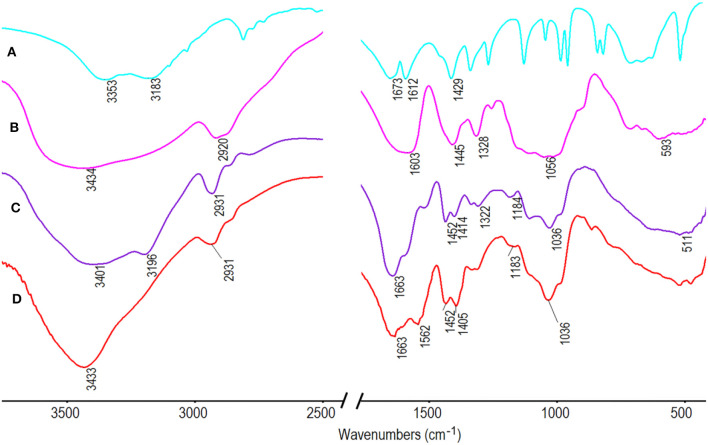
FTIR spectra of **(A)** AM; **(B)** CMC and CMC-*g*-PAM/ATP **(C)** before and **(D)** after hydrolysis.

FTIR spectra of AM, CMC, and CMC-*g*-PAM/ATP before and after hydrolysis are compared in Figure 5 to confirm the formation of the target porous adsorbent. The absorption band of CMC at 3,434 cm^−1^ (the OH stretching vibration of CMC) overlapped with the O-H stretching vibrations of the (Fe, Mg) O-H and (Al, Mg) O-H of ATP (Wang et al., 2015) after the reaction and appeared at 3,401 cm^−1^ in the spectrum in Figure 5C. The band of AM at 1,673 cm^−1^ assigned to C=O stretching vibration and the band of CMC at 1,603 cm^−1^ attributed to asymmetric stretching vibration of carboxylate overlapped and appeared at 1,663 cm^−1^ in the spectrum of CMC-*g*-PAM/ATP (Figure 5C), indicating successful grafting of the AM monomer onto the CMC (Xiao et al., 2015). The absorption band at 1,036 cm^−1^ (Figure 5C) was assigned to the Si-O-Si asymmetric stretching vibration, indicating the existence of ATP in the porous monolith. After hydrolysis, the characteristic absorption at 3,196 cm^−1^ (Figure 5C) ascribed to -NH_2_ groups disappeared, and the O-H stretching vibration presented an obvious blue shift and appeared at 3,433 cm^−1^. Furthermore, the characteristic absorption band of the C=O in amide at 1,663 cm^−1^ weakened, and the absorption peaks at 1,562 and 1,405 cm^−1^, which were assigned to asymmetric and symmetric stretching vibration of -COO^−^ groups, appeared after hydrolysis, indicating that the amide groups were successfully converted into carboxyl groups (Ghorai et al., 2014). Moreover, the absorption band of Si-O-Si asymmetric stretching vibration of ATP persisted after hydrolysis.

**Figure 6:**
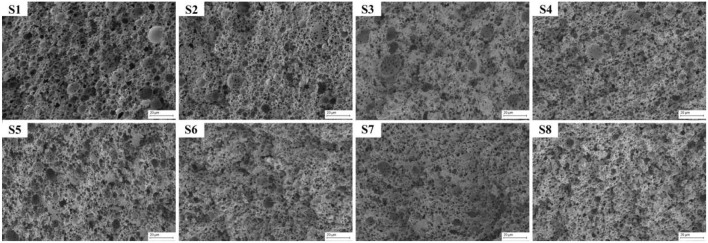
SEM images of porous CMC-*g*-PAM/ATP prepared with different oils.

The surface morphologies of the porous materials prepared by Pickering MIPEs with different vegetable oils are exhibited in Figure 6. The SEM images of eight porous materials presented similar surface morphologies, which indicated that the effect of the type of vegetable oil on the pore structure was almost negligible. All of the materials exhibit interconnected and distinct hierarchical porous structures, with both macropores and pore throats clearly visible in their SEM images. The statistical data in Table 1 indicated that the average macropore sizes of the eight materials were relatively uniform about 2.3 μm, which was consistent with the droplet size of the corresponding emulsions, indicating that there was no structural shrinkage during the polymerization and post-treatment process. In general, the macropore size of materials prepared by the high internal phase emulsion template method is about 100–700 μm (Ikem et al., 2010b). By contrast, the macropore diameter of materials prepared in this paper was greatly reduced, which could effectively improve the specific surface area of the materials.

### Adsorption Properties of CMC-g-PAM/ATP for Ce(III) and Gd(III)

**Figure 7:**
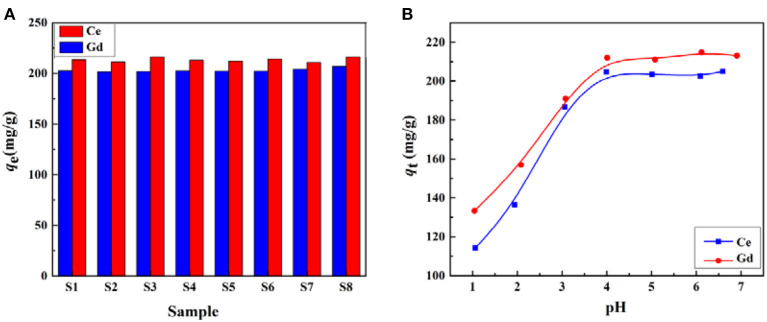
**(A)** Effect of oil phase on the adsorption capacity of the porous CMC-*g*-PAM/ATP materials for Ce(III) and Gd(III). **(B)** Effect of pH on the adsorption capacity of CMC-*g*-PAM/ATP S2 for Ce(III) and Gd(III).

The adsorption properties of CMC-*g*-PAM/ATP for Ce(III) and Gd(III) were next evaluated. As shown in Figure 7A, all adsorbents exhibited almost the same adsorption efficiency, with adsorption capacities for Ce(III) and Gd(III) of around 205 and 216 mg/g, respectively. The similar adsorption properties for Ce(III) and Gd(III) might be due to the similar chemical compositions and porous structures of the as-prepared porous monoliths. The results indicate that the Pickering MIPEs prepared in this study exhibit wide applicability and can be prepared from various kinds of vegetable oils.

#### Effect of pH on Adsorption of Ce(III) and Gd(III)

To determine the effects of pH on adsorption of Ce(III) and Gd(III), the adsorption experiments were carried out at different pH values; the results are shown in Figure 7B. When the pH value was higher than 7.0, the metal ions involved were hydrolyzed, so pH values from 1 to 7 were selected for the tests. The adsorption properties of monoliths were highly pH-dependent, and the adsorption capacity was mainly governed by the number of charged functional groups on the backbone. It was clear that the adsorption capacity of the CMC-*g*-PAM/ATP to Ce(III) and Gd(III) presented a remarkable increase with an increase in pH up to 4.0, above which the adsorption capacity became stable. This phenomenon might be attributable to the protonation of –COO^−^ under the strongly acidic conditions (Wang W. et al., 2013). The protonation would reduce the complexation ability of the adsorbent with metal ions (Ijagbemi et al., 2010). With the increase in pH value, a part of the –COOH groups transformed into –COO^−^ groups, which had stronger complexing ability with metal ions than –COOH, so the higher pH value facilitated the adsorption capacity of the adsorbents. As the initial pH increased from 4.0 to 7.0, all the carboxylic groups of the adsorbent had been dissociated and charged, and thus a constant adsorption capacity was observed.

#### Effect of Contact Time on Adsorption of Ce(III) and Gd(III)

**Figure 8:**
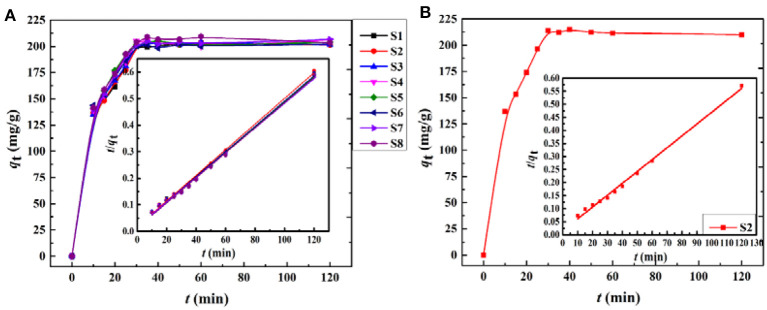
Adsorption kinetics curves of the porous CMC-*g*-PAM/ATP materials for **(A)** Ce(III) and **(B)** Gd(III) (inset plot of *t*/*q*_t_ vs. *t* for the pseudo-second-order equation).

The adsorption kinetics of the adsorbent is a significant parameter for practical application. The kinetic adsorption curves of the porous monoliths with different oil phases are shown in Figure 8. The figure demonstrates that the adsorption rate of the porous monoliths for the metal ions increased remarkably in the first 30 min and that the degree of adsorption reached a steady state after 30 min. The adsorption curves also showed that the adsorption rates of the eight materials for Ce(III) were basically consistent, and thus only S2 was selected as a representative to evaluate the adsorption rate for Gd(III). The adsorption tendency for Gd(III) showed that S2 also had a fast adsorption rate for Gd(III) and reached adsorption equilibrium by 30 min, which was similar to the adsorption behavior for Ce(III). The fast adsorption rate profited from the interconnected pores of the materials, which efficiently reduced the mass transfer resistance and exposed more binding sites for metal ions, thus improving the accessibility of metal ions to the adsorbent (Gupta et al., 2014).

In order to examine the potential rate-controlling steps during the adsorption process, pseudo-first-order (Equation 2) and pseudo-second-order (Equation 3) models (Ghaedi et al., 2015) were tested to fit the experimental data. The equations of the kinetic models are expressed as follows:

(2) $$\begin{array}{r} \text{Log}\left(qe-qt\right)=\text{log}qe-\left(k1/2.303\right)t \end{array}$$

(3) $$\begin{array}{r} t/qt=1/\left(k2\times qe2\right)+t/qe \end{array}$$

where *q*_t_ is the adsorption capacity for Ce(III) or Gd(III) at time *t*, and *q*_e_ is the equilibrium adsorption capacity. *k*_1_ (min^−1^) and *k*_2_ [g/(mg min)] are the adsorption rate constants of the pseudo-first-order and pseudo-second-order models, respectively. As shown in Table 2, the adsorption of Ce(III) and Gd(III) onto the porous monolith adsorbents followed the pseudo-second-order model rather than pseudo-first-order model by comparison with their linear correlation coefficients (*R*^2^). In addition, the calculated *q*_e_ values (*q*_e, cal_) with the pseudo-second-order kinetic model were much closer to the experimental values (*q*_e, exp_). This suggested that the adsorptions of the porous monoliths for Ce(III) and Gd(III) were probably controlled by a chemical adsorption process (Zhou S. et al., 2014; Zhou Y. et al., 2014).

**Table 2:** Adsorption kinetic parameters for adsorption of Ce(III) and Gd(III) onto CMC-*g*-PAM/ATP.

**Pseudo-first-order equation**				**Pseudo-second-order equation**				
**Sample**	***q*_**e, cal**_ (mg/g)**	***K*_**1**_ × 10^**−2**^ (min^**−1**^)**	***R*^**2**^**	***q*_**e, cal**_ (mg/g)**	***K*_**2**_ × 10^**−3**^ (g/mg min)**	***R*^**2**^**	***q*_**e, exp**_ (mg/g)**	**Metal**
S1	45.99	04.34	0.4823	212.28	1.19	0.9959	203.58	Ce(III)
S2	36.84	3.29	0.2594	207.12	1.30	0.9973	205.61	Ce(III)
S3	37.91	3.73	0.3651	211.15	1.35	0.9971	204.37	Ce(III)
S3	91.89	7.39	0.4706	214.61	1.22	0.9975	206.17	Ce(III)
S5	33.72	3.64	0.3197	211.40	1.57	0.9977	205.73	Ce(III)
S6	46.79	3.14	0.5010	210.46	1.42	0.9977	202.56	Ce(III)
S7	123.97	8.35	0.7361	215.43	1.23	0.9975	206.65	Ce(III)
S8	23.99	2.85	0.0633	211.93	1.78	0.9958	208.94	Ce(III)
S2	38.93	3.02	0.2651	220.26	1.33	0.9943	215.15	Gd(III)

#### Effect of Initial Concentration on Adsorption of Ce(III) and Gd(III)

**Figure 9:**
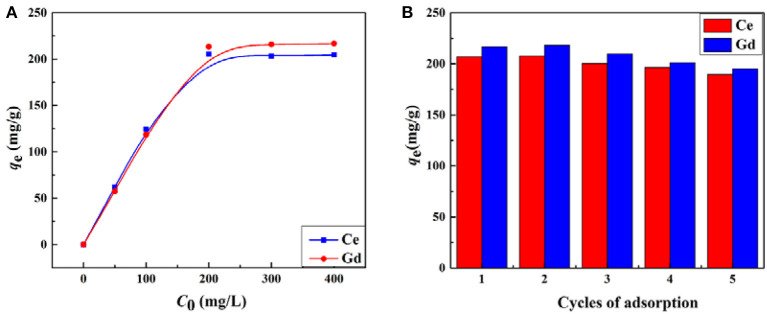
**(A)** Effect of the initial Ce(III) and Gd(III) concentrations on the adsorption capacity of porous material S2 and **(B)** the amount adsorbed for Ce(III) and Gd(III) as a function of the adsorption-desorption cycle.

The influence of the initial concentrations of metal ions on the adsorption capacity of the materials was investigated in the range of 50–400 mg/L. As shown in Figure 9A, the saturation adsorptions of the porous monolith (S2) for Ce(III) and Gd(III) were reached when the initial concentration increased to 200 mg/L, and then the increasing trend flattened. The maximum adsorption capacities for Ce(III) and Gd(III) were 205.48 and 216.73 mg/g, respectively. As the concentrations of Ce(III) and Gd(III) increased, the inside and outside concentration gradient of the material increased, thus strengthening the adsorption driving force, which was beneficial for Ce(III) and Gd(III) to overcome the transfer resistance and spread to the interior of the 3D network. Once all the adsorption sites of the adsorbent were completely occupied by metals ions, adsorption equilibrium was reached.

The adsorption process was simulated by the Langmuir (Equation 4) and Freundlich (Equation 5) isotherm models (Mittal et al., 2010; Chen et al., 2019). The respective equations are as follows.

(4) $$\begin{array}{r} \frac{C_{\text{e}}}{q_{\text{e}}}=\frac{1}{q_{\text{m}}b}+\frac{C_{\text{e}}}{q_{\text{m}}} \end{array}$$

(5) $$\begin{array}{r} \log q_{\text{e}}=\log K+\left(1/n\right)\log C_{\text{e}} \end{array}$$

where *C*_e_ (mg/L) represents the equilibrium concentration of metal ions, and *q*_e_ and *q*_m_ are the adsorption capacity (mg/g) of adsorbent at any time and in equilibrium state (mg/g), respectively. b (L/mg) is the Langmuir constant related to the affinity of binding sites (mg), which can be calculated by the slope (1/ *q*_m_) and intercept (1/ *q*_m_ × b) of plot (*C*_e_/ *q*_e_) vs. *C*_e_. *K* is a Freundlich constant related to adsorption capacity, and *n* is a dimensionless constant representing an index of adsorption intensity or surface heterogeneity. *K* and *n* can also be calculated by the slope (1/*n*) and intercept (log*K*) of plot (log*q*_e_) vs. log*C*_e_.

The related isotherm parameters and correlation coefficients (*R*^2^) were calculated and are summarized in Table 3. The fitting results proved that the relevant parameters of the Langmuir isothermal model (Ce(III): 0.9999, Gd(III): 0.9973) were much higher than those of the Freundlich isothermal model (Ce(III): 0.6563, Gd(III): 0.6148). The maximum adsorption capacities for Ce(III) and Gd(III) calculated by the Langmuir model were 205.56 and 224.52 mg/g, respectively, which were very close to the experimental values of 205.48 and 216.73 mg/g, respectively. The above analysis indicates that the Langmuir model was suitable for fitting the adsorption process of rare earth metals onto the adsorbent, suggesting that the binding sites were evenly distributed on the adsorbent surface, as these binding sites exhibited the same affinity for adsorption as a single molecular layer (Wang J. et al., 2013). Moreover, it can also be seen from Table 4 that the saturated adsorption capacities of the macroporous monolith prepared in this study were much higher than those of the adsorbents reported previously, and so was the adsorption rate.

**Table 3:** Adsorption isotherm constants of Ce(III) and Gd(III) onto CMC-*g*-PAM/ATP.

	**Langmuir model**				**Freundlich model**		
**Metal**	***q*_**e, exp**_ (mg/g)**	***q*_**m**_ (mg/g)**	***b* (L/mg)**	***R*^**2**^**	**K**	***n***	***R*^**2**^**
Ce^3+^	205.48	205.56	0.7734	0.9999	86.68	5.57	0.6563
Gd^3+^	216.73	224.52	0.1489	0.9973	56.73	3.58	0.6148

**Table 4:** Comparison of adsorption capacities (*q*_m_, mg/g) and rates (T, min) of various adsorbents for Ce(III) and Gd(III).

**Adsorbent**	**Ce(III)**		**Gd(III)**		**References**
	***q*_**m**_ (mg/g)**	**T (min)**	***q*_**m**_ (mg/g)**	**T (min)**	
Zn/Al LDH-intercalated cellulose	96.25	10	−	−	Iftekhar et al., 2017
Corn style	180.20	240	−	−	Jaya et al., 2014
Prawn carapace	218.30	360	−	−	Jaya et al., 2014
Mesoporous conjugate adsorbent	192.31	25	−	−	Awual et al., 2013
Loofah fiber-*g*-acrylic acid (LF-AA)	527.5	120	−	−	Liu et al., 2017
Wheat straw	298.60	100	−	−	Zhou et al., 2016
Poly(allylamine)/silica composite	111.8	60	−	−	Zhou S. et al., 2014
Colloidal graphene oxide	−	−	286.86	30	Chen et al., 2014
1,2-HOPO-SAMMS	−	−	80	30	Yantasee et al., 2010
Cross-linked polyvinyl amidoxime fiber	−	−	14.83	150	Yang et al., 2018
By-pass cement	−	−	100		Ali et al., 2011
Polyethyleneglycol (phosphomolybdate and tungstate)	−	−	57	−	Zhang et al., 2009
CTS-*g*-(AA-*co*-SS)/ISC hydrogel	174.05	20	223.79	20	Wang et al., 2017a
Kenaf cellulose-based poly(hydroxamic acid)	245	−	220	−	Rahman et al., 2017
CMC-*g*-PAM/ATP	205.48	30	216.73	30	This study

#### Reusability of CMC-g-PAM/ATP for Ce(III) and Gd(III)

Reusability is an important parameter for evaluating the properties of an adsorbent. In this section, S2 was selected to investigate the regeneration performance. Given that the prepared porous adsorbent contains a large number of carboxyl groups, which are sensitive to H^+^, hydrochloric acid (0.5 mol/L) was selected as the eluent for desorption of Ce(III) and Gd(III) from CMC-*g*-PAM/ATP after adsorption. After the desorption experiment, the material was activated with 0.1 mol/L NaOH and used for the next adsorption process. As shown in Figure 9B, the adsorption capacities of the adsorbent in the second cycle were a little higher than the initial capacities, suggesting that some adsorption sites can be created during the regeneration process; a similar phenomenon was also found in earlier studies (Zhu et al., 2015). After the second cycle, there was only a subtle decrease in the adsorption capacities for Ce(III) and Gd(III). Furthermore, the prepared porous material maintained relatively high adsorption capacities to Ce(III) and Gd(III) during the whole consecutive adsorption-desorption processes, indicating that the as-prepared hydrogel CMC-*g*-PAM/ATP exhibited excellent reusability.

## Conclusion

A convenient, economical, and environmentally friendly strategy was developed for the preparation of interconnected porous adsorbent of CMC-*g*-PAM/ATP with vegetable oil-in-water Pickering-MIPE templates that was stabilized by raw ATP and a trace amount of T-20. The stability of the emulsion could be facilely modulated by the emulsification rate and duration. The type of vegetable oil has no significant influence on either the properties of the emulsion or the porous structure of the material. SEM images indicated that all materials derived via the Pickering-MIPE template method exhibited an excellent interconnected hierarchical porous structure that was not inferior to that prepared with HIPE. Due to the highly permeable porous structure and abundant functional groups, the materials prepared presented promising potential as excellent adsorbents for the rapid extraction of Ce(III) and Gd(III) from aqueous solution. Furthermore, the materials still exhibited excellent reusability after five consecutive adsorption-desorption processes. This work can be taken as a new contribution to efforts to develop a new pathway to construct porous adsorbents for the adsorption and enrichment of REEs.

## Data Availability Statement

All datasets generated for this study are included in the article/supplementary material.

## Author Contributions

FW contributed to the experimental process and data analysis, wrote the paper, and drew all the figures. YZ contributes to the data analysis and revision of the paper. AW contributed to the experimental design and revision of the paper.

## Conflict of Interest

The authors declare that the research was conducted in the absence of any commercial or financial relationships that could be construed as a potential conflict of interest.
